# Nucleus Accumbens Microcircuit Underlying D2-MSN-Driven Increase in Motivation

**DOI:** 10.1523/ENEURO.0386-18.2018

**Published:** 2018-05-17

**Authors:** Carina Soares-Cunha, Bárbara Coimbra, Ana Verónica Domingues, Nivaldo Vasconcelos, Nuno Sousa, Ana João Rodrigues

**Affiliations:** 1 Life and Health Sciences Research Institute (ICVS), School of Medicine, University of Minho, Braga, 4710-057, Portugal; 2 ICVS/3B’s–PT Government Associate Laboratory, Braga/Guimarães, 4710-057, Portugal; 3 Departamento de Física, Universidade Federal de Pernambuco, Recife, Pernambuco 50670-901, Brazil

**Keywords:** basal ganglia, medium spiny neurons, motivation, nucleus accumbens, optogenetics, reward

## Abstract

The nucleus accumbens (NAc) plays a central role in reinforcement and motivation. Around 95% of the NAc neurons are medium spiny neurons (MSNs), divided into those expressing dopamine receptor D1 (D1R) or dopamine receptor D2 (D2R). Optogenetic activation of D2-MSNs increased motivation, whereas inhibition of these neurons produced the opposite effect. Yet, it is still unclear how activation of D2-MSNs affects other local neurons/interneurons or input terminals and how this contributes for motivation enhancement. To answer this question, in this work we combined optogenetic modulation of D2-MSNs with *in loco* pharmacological delivery of specific neurotransmitter antagonists in rats. First, we showed that optogenetic activation of D2-MSNs increases motivation in a progressive ratio (PR) task. We demonstrated that this behavioral effect relies on cholinergic-dependent modulation of dopaminergic signalling of ventral tegmental area (VTA) terminals, which requires D1R and D2R signalling in the NAc. D2-MSN optogenetic activation decreased ventral pallidum (VP) activity, reducing the inhibitory tone to VTA, leading to increased dopaminergic activity. Importantly, optogenetic activation of D2-MSN terminals in the VP was sufficient to recapitulate the motivation enhancement. In summary, our data suggests that optogenetic stimulation of NAc D2-MSNs indirectly modulates VTA dopaminergic activity, contributing for increased motivation. Moreover, both types of dopamine receptors signalling in the NAc are required in order to produce the positive behavioral effects.

## Significance Statement

The nucleus accumbens (NAc) is a key brain region of the reward system and is crucial for motivation. We showed that activation of NAc D2-expressing neurons enhances motivation by modulating ventral tegmental area (VTA) dopaminergic activity via ventral pallidum (VP) inhibition. The behavioral effect was dependent on local cholinergic-dependent dopamine release by VTA terminals that required D1 and D2 dopamine receptors (D1R and D2R, respectively) in the NAc. This study reveals for the first time how D2-MSN stimulation can modulate downstream regions and local microcircuit to increase motivation.

## Introduction

Dopaminergic projections from the ventral tegmental area (VTA) to the nucleus accumbens (NAc) have been classically described as the core of the reward circuit (Wise, 2004). Evidence in animal models and humans showed that the motivational aspects of reward processing are greatly mediated by these projections (Wise, 1998; Kelley and Berridge, 2002; Hyman et al., 2006; Bailey et al., 2016). The NAc contains 95% of medium spiny neurons (MSNs), that are typically divided into those that express dopamine receptor D1 (D1R, D1-MSNs), and those that express dopamine receptor D2 (D2R, D2-MSNs). In addition to dopaminergic inputs from the VTA, these MSNs receive dense monosynaptic glutamatergic innervation from the medial prefrontal cortex, hippocampus and amygdala (Haber, 2003). These MSNs project directly to the VTA through the direct pathway, mediated exclusively by D1-MSNs, or indirectly via the ventral pallidum (VP; both D1- and D2-MSNs; Lu et al., 1998; Zhou et al., 2003; Kupchik et al., 2015). Additionally, MSNs are known to synapse within each other (Sesack and Pickel, 1990; Dobbs et al., 2016), maintaining GABAergic accumbal activity under a balanced control.

The remaining 5% of NAc neurons are local interneurons, that include large tonically active cholinergic interneurons (CINs), fast spiking (FS) GABAergic interneurons, low-threshold spiking (LTS) interneurons (Soares-Cunha et al., 2016b), as well as less explored subtypes, namely tyrosine hydroxylase interneurons (Ibáñez-Sandoval et al., 2010, 2015) and calretinin interneurons (Tepper and Bolam, 2004). Importantly, both cholinergic and GABAergic interneurons play a crucial role in NAc activity and response to salient stimuli and modulate reward-dependent behaviors (Tepper and Bolam, 2004; Lim et al., 2014).

In the past years, compelling data supported a role for D1-MSNs in positive reinforcement, while D2-MSNs have been mostly associated with aversion. Nonetheless, recent data emerged in opposition to this dichotomy; whereas the division of direct and indirect neurons based on the respective expression of D1R and D2R in dorsal striatum appears to be precise, in the NAc the indirect pathway contains a mixture of D1-MSNs and D2-MSNs (Lobo et al., 2010; Kravitz et al., 2012). This implies that both NAc D1- and D2-MSNs can inhibit or disinhibit thalamic activity, with clear repercussions in behavior. In agreement with this view, a previous study showed that activation of either NAc D1- or D2-MSNs is sufficient to increase motivation in a progressive ratio (PR) task (Soares-Cunha et al., 2016a). In the same direction, in the ventrolateral striatum, both D1- and D2-MSNs are activated at the trial start cue in the PR test and inhibition of either population immediately after the cue resulted in decreased motivation (Natsubori et al., 2017).

These seminal findings showed that D2-MSNs play a more pro-motivation/reward role than initially anticipated and suggest that the prevailing notion of a functional segregation of MSNs should be reconsidered. Yet, it is still unclear how activation of D2-MSNs affects other local neurons/interneurons and downstream regions and how this contributes for motivation enhancement. Therefore, we combined optogenetic activation of NAc D2-MSNs with *in loco* pharmacological delivery of specific antagonists to identify the contribution of different NAc inputs and neuronal populations for motivational drive.

## Materials and Methods

### Animals

Male Wistar Han rats (two to three months old at the beginning of the tests) were used. Animals were maintained under standard laboratory conditions: 12/12 h light/dark cycle (lights on from 8 A.M. to 8 P.M.) and room temperature of 21 ± 1°C, with relative humidity of 50–60%; rats were individually housed after optical fiber implantation; standard diet (4RF21, Mucedola SRL) and water were given ad libitum, until the beginning of the behavioral experiments, in which animals switched to food restriction to maintain 85% of initial body weight.

Behavioral manipulations occurred during the light period of the light/dark cycle. Health monitoring was performed according to FELASA guidelines (Nicklas et al., 2002). All procedures were conducted in accordance with European Regulations (European Union Directive 2010/63/EU). Animal facilities and animals’ experimenters were certified by the National regulatory entity, Direção-Geral de Alimentação e Veterinária (DGAV). All protocols were approved by the Ethics Committee of the Life and Health Sciences Research Institute (ICVS) and by DGAV.

### Experimental design

Group I of animals (n_D2-ChR2_ = 10, n_D2-eYFP_ = 7), which received intracranial viral injection and optical fiber placement in the NAc, performed the PR test (described in behavior section” throughout) and were killed 90 min after the beginning of the last PR session for c-fos analysis (Extended Data Fig. 1-1*A*).

Group II of animals (n_D2-ChR2_ = 8, n_D2-eYFP_ = 7), which received intracranial viral injection and hybrid cannula (optics and fluid) placement in the NAc, performed the PR test (described below) and performed two additional PR sessions with antagonist injections. On day 1, half of the animals received antagonist injection and the other half received vehicle injection. On day 2, animals receiving drug on the first day received vehicle and vice versa. All animals were treated with vehicle and drug. After behavioral performance, all rats were killed, and cannula placement and viral expression were confirmed (Extended Data Fig. 1-1*B*).

Group III of animals (n_D2-ChR2 NAc-VP_ = 8, n_D2-eYFP NAc-VP_ = 6), which received intracranial viral injection in the NAc and optical fiber placement in the VP, performed the PR test (described below; Extended Data Fig. 1-1*C*).

Group IV of animals (n_D2-ChR2_ = 4) was injected with ChR2 in the NAc, and after three weeks to allow viral expression, *in vivo* single unit electrophysiological recordings were performed (Extended Data Fig. 1-1*D*).

### Behavior

#### Subjects and apparatus

Rats were habituated to 45 mg of food pellets (F0021; Bio-Serv), which were used as reward during the behavioral protocol, 1 d before training initiation. Behavioral sessions were performed in operant chambers (Med Associates) that contained a central, recessed magazine to provide access to 45 mg of food pellets (Bio-Serve), two retractable levers with cue lights located above them that were located on each side of the magazine. Chamber illumination was obtained through a 2.8-W, 100-mA light positioned at the top-center of the wall opposite to the magazine. The chambers were controlled by a computer equipped with the Med-PC software (Med Associates).

#### PR schedule of reinforcement

All training sessions started with illumination of the house light that remained until the end of the session. On the first training session [continuous reinforcement (CRF) sessions] one lever was extended. The lever would remain extended throughout the session, and a single lever press would deliver a food pellet (maximum of 50 pellets earned within 30 min). In some cases, food pellets were placed on the lever to promote lever pressing. After successful completion of the CRF training, rats were trained to lever press on the opposite lever using the same training procedure. In the four following days, the side of the active lever was alternated between sessions. Then, rats were trained to lever press one time for a single food pellet in a fixed ratio (FR) schedule consisting in 50 trials in which both levers are presented, but the active lever is signaled by the illumination of the cue light above it. FR sessions began with extension of both levers (active and inactive) and illumination of the house light and the cue light over the active lever. Completion of the correct number of lever press led to a pellet delivery, retraction of the levers and the cue light turning off for a 20-s intertrial interval (ITI). Rats were trained first with one lever active and then with the opposite lever active in separate sessions (in the same day). In a similar manner, rats were then trained using an FR4 reinforcement schedule for 4 d and a FR8 for 1 d. On the test day, rats were exposed to PR or FR experimental sessions (one session per day) according to the following schedule: day 1, FR4; day 2, PR (optical stimulation); day 3, FR4; day 4, PR (no optical stimulation). PR sessions were identical to FR4 sessions except that the operant requirement on each trial (T) was the integer (rounded down) of 1.4^(T–1)^ lever presses, starting at 1 lever press. PR sessions ended after 15 min elapsed without completion of the response requirement in a trial.

Before the PR session, rats were connected to an opaque optical fiber, through previously implanted cannula guide placed in the NAc. At the beginning of each trial of the PR session with optical stimulation, when the retractable levers are exposed to the animal together with the cue light, animals received an optical stimulation. After basal assessment of PR (one session with optical stimulation and one session without), all animals performed seven additional sessions (with one-week interval and one FR4 reminder session before PR test) with optical stimulation and local pharmacological administration of receptors antagonist (Extended Data Fig. 1-1).

Optical stimulation was performed as follows: 473 nm; frequency of 40 Hz; 12.5-ms pulses over 1 s; 10 mW at the tip of the implanted fiber.

#### Constructs and virus preparation

eYFP or hChR2(H134R)-eYFP were cloned under the control of the D2R minimal promoter region as described before (Soares-Cunha et al., 2016a; Zalocusky et al., 2016). Constructs were packaged in AAV5 serotype by the University of North Carolina at Chapel Hill (UNC) Gene Therapy Center Vector Core (UNC). AAV5 vector titters were 3.7–6 × 10^12^ viral molecules/ml as determined by dot blot.

#### Surgery and cannula implantation

Rats were anesthetized with 75 mg kg^−1^ ketamine (Imalgene, Merial) plus 0.5 mg kg^−1^ medetomidine (Dorbene, Cymedica). Virus was unilaterally injected into the NAc; coordinates from bregma, according to (Paxinos and Watson, 2005): +1.2 mm anteroposterior (AP), +1.2 mm mediolateral (ML), and −6.5 mm dorsoventral (DV; D2-ChR2 group and D2-eYFP control group). Rats that performed the PR with only optical stimulation were implanted with an optic fiber (200 μm in diameter) attached to a 2.5-mm ferrule (Thorlabs), and rats that performed the PR test with both optical stimulation and local administration of antagonists were implanted with opto-fluid cannulas (Doric Lenses) using the injection coordinates (except for the DV: −6.4 mm) that were secured to the skull using 2.4-mm screws (Bilaney) and dental cement (C&B kit, Sun Medical).

For NAc terminal stimulation in the VP, virus was injected as above but rats were implanted with an optic fiber in the VP (coordinates from bregma) −0.1 mm AP, +2.4 mm ML, and −7 mm DV (D2-ChR2 NAc-VP group and D2-eYFP NAc-VP control group).

Rats were allowed to recover for two weeks before initiation of the behavioral trainings.

### *In vivo* single-cell electrophysiology

Three weeks postsurgery, D2-ChR2 rats (*n* = 4) were anaesthetized with urethane (1.44 g kg^−1^, Sigma). The total dose was administered in three separate intraperitoneal injections, 15 min apart. Adequate anesthesia was confirmed by the lack of withdrawal responses to hindlimb pinching. A recording electrode coupled with a fiber optic patch cable (Thorlabs) was placed in the NAc (coordinates from bregma: +1.2 mm AP, +1.2 mm ML, and −6.0 to −7.0 mm DV), using a stereotaxic frame (David Kopf Instruments) with nontraumatic ear bars (Stoeling). Other recording electrodes with fiber optic attached were placed in the VP (coordinates from bregma: 0 to −0.12 mm AP, +2.3 to +2.5 mm ML, and −7 to −7.6 mm DV) and in the VTA (coordinates from bregma: −5.3 mm AP, +0.9 mm ML, and −7.5 to −8.3 mm DV).

Single neuron activity was recorded extracellularly with a tungsten electrode (tip impedance 5–10 Mat 1 kHz) and data sampling was performed using a CED Micro1401 interface and Spike2 software (Cambridge Electronic Design). The DPSS 473 nm laser system, controlled by a stimulator (Master-8, AMPI) was used for intracranial light delivery. Optical stimulation was performed as follows: 473 nm; frequency of 40 Hz; 12.5-ms pulses over 1 s, 10 mW.

Firing rate histograms were calculated for the baseline (10 s before stimulation), stimulation period and after stimulation period (10 s after the end of stimulation). Spike latency was determined by measuring the time between half-peak amplitude for the falling and rising edges of the unfiltered extracellular spike.

NAc neurons were classified according to previous descriptions (Jin et al., 2014; Vicente et al., 2016). In short, fast-spiking interneurons (FSIs), putative parvalbumin-containing neurons (pFSs), were identified has having a waveform half-width of less that 100 μs and a baseline firing rate higher that 10 Hz; tonically active putative CINs (pCINs) were identified as those with a wave form half-width bigger that 300 μs. Putative MSNs (pMSNs) were identified as those with baseline firing rate lower that 5 Hz and that do not met the wave form criteria for pCIN or pFS neurons.

VP GABAergic neurons were identified as those having a baseline firing rate between 0.2 and 18.7 Hz (Richard et al., 2016). Other nonidentified neurons (corresponding to less that 5% of recorded cells) were excluded from the analysis.

Single units in the VTA were separated into those putative dopaminergic (pDAergic) and putative GABAergic (pGABAergic). This classification was based on firing rate and wave form duration (Ungless et al., 2004; Ungless and Grace, 2012; Totah et al., 2013). Cells presenting baseline firing rate lower that 10 Hz and a wave form duration higher than 1.5 ms were considered pDAergic neurons. Cells presenting baseline firing rate higher than 10 Hz and wave form duration lower than 1.5 ms were classified as pGABAergic. Other single units that did not fit in any classification (<5% of recorded cells) were excluded from the analysis.

### Immunofluorescence (IF)

Ninety minutes after initiation of the PR test, rats were deeply anesthetized with pentobarbital (Eutasil) and were transcardially perfused with 0.9% saline followed by 4% paraformaldehyde. Brains were removed and postfixed in 4% paraformaldehyde. Coronal vibratome sections (50 μm) were incubated with mouse anti-D2R (1:500, catalog #sc-5303, RRID: AB_668816, Santa Cruz Biotechnology), rabbit anti-c-fos (1:1000, Merck Millipore catalog #Ab-5, RRID: AB_2314042), goat or mouse anti-GFP (1:500, Abcam catalog #ab6673, RRID: AB_305643; or Abcam catalog #ab1218, RRID: AB_298911), mouse anti-D1R (1:100, Novus catalog #NB110-60017, RRID: AB_905382), and goat anti-ChAT (1:750, Millipore catalog #AB144P, RRID: AB_2079751). Appropriate secondary fluorescent antibodies were used (1:500, Invitrogen; catalog #A-21206, RRID:AB_141708; catalog #R37119, RRID:AB_2556547; catalog #A-21202, RRID:AB_141607; catalog #R37114, RRID:AB_2556542; catalog #A-11055, RRID:AB_142672). Finally, all sections were stained with 4’,6-diamidino-2-phenylindole (DAPI; 1 mg ml^−1^). Anti-D1R and anti-D2R antibodies were previously validated (Luedtke et al., 1999; Basu et al., 2004; Luessen et al., 2016; Extended Data Fig. 5-1).

For each brain region, countings were performed in five distinct 50-μm sections. Images were collected and analyzed by confocal microscopy (Olympus FluoViewTMFV1000). Cell counts were normalized to the area of the brain region.

### Drugs

All drugs were delivered 10 min before animals performed the PR test, through an opto-fluid system chronically implanted in the NAc. Injections were performed using a 5-μl gastight seringe (Hamilton), attached to the implanted injection cannula of the rats through 22-gauge tubing, at a constant rate of 1 μl/min.

The drugs used in experimental procedures were: R(+)-SCH-23390 hydrochloride (D1R antagonist, 0.25 μg in 0.5 μl of saline, Sigma); (S)-(-)-sulpiride (D2R antagonist, 0.2 μg in 1 μl, Sigma); scopolamine hydrobromide [muscarinic acetylcholine receptor (mAChR) antagonist, 25 μg in 1 μl, Sigma]; mecamylamine hydrochloride (nAChR antagonist, 22.5 μg in 1 μl, Sigma); Ddihydro-β-erythroidine hydrobromide (DHβE, α4-nAChR antagonist, 0.7 μg in 1 μl, Tocris); CGP-55845 hydrochloride (GABA(B) receptor antagonist, 44 ng in 0.5 μl, Sigma); 1(S),9(R)-(-)-bicuculine methobromide (GABA(A) receptor antagonist, 75 ng in 0.5 μl, Sigma).

### Statistical analysis

Normality tests were performed for all data analyzed, as well as outlier analysis using Tukey’s test. Statistical analysis between two groups was made using two-tailed Student’s *t* test (unpaired *t* test for comparison between two groups; paired *t* test for comparison within the same group). One- or two-way ANOVA was used when appropriate. Bonferroni’s *post hoc* multiple comparisons were used for group differences determination. Statistical results are displayed in Table 1. Results are presented as mean ± SEM. All statistical analysis was performed using GraphPad Prism (v7.0), and results were considered significant for *p* ≤ 0.05.

**Table 1.** Statistical table

Figure	Data structure	Sample size	Type of test	Statistics
1*F*	Normal distribution	23 cells from four rats	One-way ANOVA	*F*_(2,48)_ = 74.7, *p* < 0.000
1*H*	Normal distribution	n_D2-eYFP_ = 7; n_D2-ChR2_ = 10	Two-way ANOVA	*F*_(1,13)_ = 0.43, *p* = 0.322
1*I*	Normal distribution	n_D2-eYFP_ = 7; n_D2-ChR2_ = 10	Two-way ANOVA	*F*_(3,30)_ = 124.8, *p* < 0.000
1*J*	Normal distribution	n_D2-eYFP_ = 7; n_D2-ChR2_ = 10	Unpaired *t* test, two tailed	*t*_(13)_ = 7.7, *p* < 0.000
1*K*	Normal distribution	n_D2-eYFP_ = 7; n_D2-ChR2_ = 10	Two-way ANOVA	Laser effect: *F*_(1,13)_ = 47.3, *p* < 0.000 Group effect: *F*_(1,13)_ = 7.9, *p* < 0.000 Bonferroni post test: D2-ChR2 ON vs D2-ChR2 OFF: *p* < 0.001
1*L*	Normal distribution	n_D2-eYFP_ = 7; n_D2-ChR2_ = 10	Unpaired *t* test, two tailed	*t*_(13)_ = 1.3, *p* = 0.1380
1*M*	Normal distribution	n_D2-eYFP_ = 7; n_D2-ChR2_ = 10	Unpaired *t* test, two tailed	*t*_(13)_ = 0.7, *p* = 0.4719
1*N*	Normal distribution	n_D2-eYFP_ = 7; n_D2-ChR2_ = 10	Unpaired *t* test, two tailed	*t*_(13)_ = 1.0, *p* = 0.3124
2*B*	Normal distribution	n_D2-eYFP veh_ = 7; n_D2-eYFP GABAA antag_ = 7; n_D2-ChR2 veh_ = 8; n_D2-ChR2 GABAA antag_ = 8	Two-way ANOVA	Treatment effect: *F*_(1,13)_ = 0.1, *p* = 0.117 Group effect: *F*_(1,13)_ = 118.8, *p* < 0.000 Bonferroni post test: D2-ChR2 vehicle vs D2-ChR2 GABA(A) antag: *p* = 0.787
2*C*	Normal distribution	n_D2-eYFP veh_ = 7; n_D2-eYFP GABAB antag_ = 7; n_D2-ChR2 veh_ = 8; n_D2-ChR2 GABAB antag_ = 8	Two-way ANOVA	Treatment effect: *F*_(1,13)_ = 30.7, *p* < 0.000 Group effect: *F*_(1,13)_ = 193, *p* < 0.000 Bonferroni post test: D2-eYFP vehicle vs D2-eYFP GABA(B) antag: *p* = 0.07 D2-ChR2 vehicle vs D2-ChR2 GABA(B) antag: *p* < 0.000
2*D*	Normal distribution	n_D2-eYFP veh_ = 7; n_D2-eYFP mAChR+nAChR antag_ = 7; n_D2-eYFP mAChR antag_ = 7; n_D2-eYFP nAChR antag_ = 7; n_D2-ChR2 veh_ = 8; n_D2-ChR2 mAChR+nAChR antag_ = 8; n_D2-ChR2 mAChR antag_ = 8; n_D2-ChR2 nAChR antag_ = 8	Two-way ANOVA	Treatment effect: *F*_(3,39)_ = 4.3, *p* = 0.001 Bonferroni post test: D2-ChR2 vehicle vs D2-ChR2 mAChR+nAChR antag: *p* < 0.000 D2-ChR2 vehicle vs D2-ChR2 nAChR antag: *p* < 0.000
2*E*	Normal distribution	n_D2-eYFP veh_ = 7; n_D2-eYFP α4*-nAChR antag_ = 7; n_D2-ChR2 veh_ = 8; n_D2-ChR2 α4*-nAChR antag_ = 8	Two-way ANOVA	Treatment effect: *F*_(1,13)_ = 43.0, *p* < 0.000 Bonferroni post test: D2-ChR2 vehicle vs D2-ChR2 α4 antag: *p* < 0.000
2*F*	Normal distribution	n_D2-eYFP veh_ = 7; n_D2-eYFP D1R antag_ = 7; n_D2-ChR2 veh_ = 8; n_D2-ChR2 D1R antag_ = 8	Two-way ANOVA	Treatment effect: D1R antag: *F*_(1,13)_ = 43.7, *p* < 0.000 Bonferroni post test: D2-eYFP vehicle vs D2-eYFP D1R antag: *p* = 0.047 D2-ChR2 vehicle vs D2-ChR2 D1R antag: *p* < 0.000
2*G*	Normal distribution	n_D2-eYFP veh_ = 7; n_D2-eYFP D2R antag_ = 7; n_D2-ChR2 veh_ = 8; n_D2-ChR2 D2R antag_ = 8	Two-way ANOVA	Treatment effect: D2R antag: *F*_(1,13)_ = 34.8, *p* < 0.000 Bonferroni post test: D2-eYFP vehicle vs D2-eYFP D2R antag: *p* = 0.013 D2-ChR2 vehicle vs D2-ChR2 D2R antag: *p* < 0.000
3*B*	Normal distribution	n_D2-ChR2_ = 8; n_D2-eYFP_ = 7	Unpaired *t* test, two tailed	D2-ChR2 vs D2-eYFP rats: *t*_(13)_ = 12.0, *p* < 0.000
3*B*	Normal distribution	n_D2-ChR2_ = 8	Paired *t* test, two tailed	Stimulated vs contralateral side: *t*_(7)_ = 7.4, *p* = 0.0002
3*C*	Normal distribution	n_D2-ChR2_ = 8; n_D2-eYFP_ = 7	Unpaired *t* test, two tailed	D2-ChR2 vs D2-eYFP rats: *t*_(13)_ = 3.7, *p* = 0.0028
3*C*	Normal distribution	n_D2-ChR2_ = 8	Paired *t* test, two tailed	Stimulated vs contralateral side: *t*_(7)_ = 3.3, *p* = 0.0011
3*D*	Normal distribution	n_D2-ChR2_ = 8; n_D2-eYFP_ = 7	Unpaired *t* test, two tailed	D2-ChR2 vs D2-eYFP rats: *t*_(13)_ = 3.7, *p* < 0.000
3*D*	Normal distribution	n_D2-ChR2_ = 8	Paired *t* test, two tailed	Stimulated vs contralateral side: *t*_(7)_ = 4.0, *p* = 0.0033
3*F*	Normal distribution	n_D2-ChR2_ = 8; n_D2-eYFP_ = 7	Unpaired *t* test, two tailed	D2-ChR2 vs D2-eYFP rats: *t*_(13)_ = 3.3, *p* < 0.000
3*F*	Normal distribution	n_D2-ChR2_ = 8	Paired *t* test, two tailed	Stimulated vs contralateral side: *t*_(7)_ = 4.4, *p* = 0.0024
3*G*	Normal distribution	n_D2-ChR2_ = 8; n_D2-eYFP_ = 7	Unpaired *t* test, two tailed	D2-ChR2 vs D2-eYFP rats: *t*_(13)_ = 0.3, *p* = 0.418
3*G*	Normal distribution	n_D2-ChR2_ = 8	Paired *t* test, two tailed	Stimulated vs contralateral side: *t*_(7)_ = 0.1, *p* = 0.9099
3*I*	Normal distribution	n_D2-ChR2_ = 8; n_D2-eYFP_ = 7	Unpaired *t* test, two tailed	D2-ChR2 vs D2-eYFP rats: *t*_(13)_ = 2.3, *p* = 0.039
3*I*	Normal distribution	n_D2-ChR2_ = 8	Paired *t* test, two tailed	Stimulated vs contralateral side: *t*_(7)_ = 1.2, *p* = 0.238
4*B*	Normal distribution	30 cells from four rats	One-way ANOVA	*F*_(2,87)_ = 10.4, *p* < 0.000
4*E*	Normal distribution	29 pDAergic cells from four rats; three pGABAergic cells from four rats	One-way ANOVA	pDAergic: *F*_(2,34)_ = 17.4, *p* < 0.000 pGABAergic: *F*_(2,8)_ = 2.7, *p* = 0.1343
4*I*	Normal distribution	n_D2-ChR2 NAc-VP_ = 8, n_D2-eYFP NAc-VP_ = 4	Unpaired *t* test, two tailed	*t*_(11)_ = 10.7, *p* < 0.000
4*J*	Normal distribution	n_D2-ChR2 NAc-VP_ = 8,	Paired *t* test, two tailed	*t*_(4)_ = 10.2, *p* < 0.000
4*K*	Normal distribution	n_D2-ChR2 NAc-VP_ = 8, n_D2-eYFP NAc-VP_ = 4	Unpaired *t* test, two tailed	*t*_(12)_ = 1.7, *p* = 0.112
2-2*A*	Normal distribution	n_D2-eYFP veh_ = 7; n_D2-eYFP GABAA antag_ = 7; n_D2-ChR2 veh_ = 8; n_D2-ChR2 GABAA antag_ = 8	Two-way ANOVA	Bonferroni post test: D2-eYFP vehicle vs D2-eYFP antag: *p* = 0.4831 D2-ChR2 vehicle vs D2-ChR2 antag: *p* = 0.7434
2-2*B*	Normal distribution	n_D2-eYFP veh_ = 7; n_D2-eYFP GABAB antag_ = 7; n_D2-ChR2 veh_ = 8; n_D2-ChR2 GABAB antag_ = 8	Two-way ANOVA	Bonferroni post test: D2-eYFP vehicle vs D2-eYFP antag: *p* = 0.7334 D2-ChR2 vehicle vs D2-ChR2 antag: *p* = 0.9332
2-2*C*	Normal distribution	n_D2-eYFP veh_ = 7; n_D2-eYFP mAChR+nAChR antag_ = 7; n_D2-eYFP mAChR antag_ = 7; n_D2-eYFP nAChR antag_ = 7; n_D2-ChR2 veh_ = 8; n_D2-ChR2 mAChR+nAChR antag_ = 8; n_D2-ChR2 mAChR antag_ = 8; n_D2-ChR2 nAChR antag_ = 8	Two-way ANOVA	Bonferroni post test: D2-eYFP vehicle vs D2-eYFP mAChR+nAChR antag: *p* = 0.9994 D2-ChR2 vehicle vs D2-ChR2 mAChR+nAChR antag: *p* = 0.9883 D2-eYFP vehicle vs D2-eYFP mAChR antag: *p* = 0.9994 D2-ChR2 vehicle vs D2-ChR2 mAChR antag: *p* = 0.9883 D2-eYFP vehicle vs D2-eYFP nAChR antag: *p* = 0.4483 D2-ChR2 vehicle vs D2-ChR2 nAChR antag: *p* = 0.9187
2-2*D*	Normal distribution	n_D2-eYFP veh_ = 7; n_D2-eYFP α4*-nAChR antag_ = 7; n_D2-ChR2 veh_ = 8; n_D2-ChR2 α4*-nAChR antag_ = 8	Two-way ANOVA	Bonferroni post test: D2-eYFP vehicle vs D2-eYFP antag: *p* = 0.4489 D2-ChR2 vehicle vs D2-ChR2 antag: *p* = 0.7023
2-2*E*	Normal distribution	n_D2-eYFP veh_ = 7; n_D2-eYFP D1R antag_ = 7; n_D2-ChR2 veh_ = 8; n_D2-ChR2 D1R antag_ = 8	Two-way ANOVA	Bonferroni post test: D2-eYFP vehicle vs D2-eYFP antag: *p* = 0.0144 D2-ChR2 vehicle vs D2-ChR2 antag: *p* = 0.0842
2-2*F*	Normal distribution	n_D2-eYFP veh_ = 7; n_D2-eYFP D2R antag_ = 7; n_D2-ChR2 veh_ = 8; n_D2-ChR2 D2R antag_ = 8	Two-way ANOVA	Bonferroni post test: D2-eYFP vehicle vs D2-eYFP antag: *p* = 0.9999 D2-ChR2 vehicle vs D2-ChR2 antag: *p* = 0.2308
4-1*B*	Normal distribution	n_D2-ChR2 NAc-VP_ = 8, n_D2-eYFP NAc-VP_ = 6	Two-way ANOVA	Group effect: *F*_(1,72)_ = 0.0, *p* = 0.856
4-1*C*	Normal distribution	n_D2-ChR2 NAc-VP_ = 8, n_D2-eYFP NAc-VP_ = 6	Two-way ANOVA	Day of training effect: *F*_(3,24)_ = 180.4, *p* < 0.000

## Results

### Optogenetic stimulation of NAc D2-MSNs increases motivation

**Figure 1.**
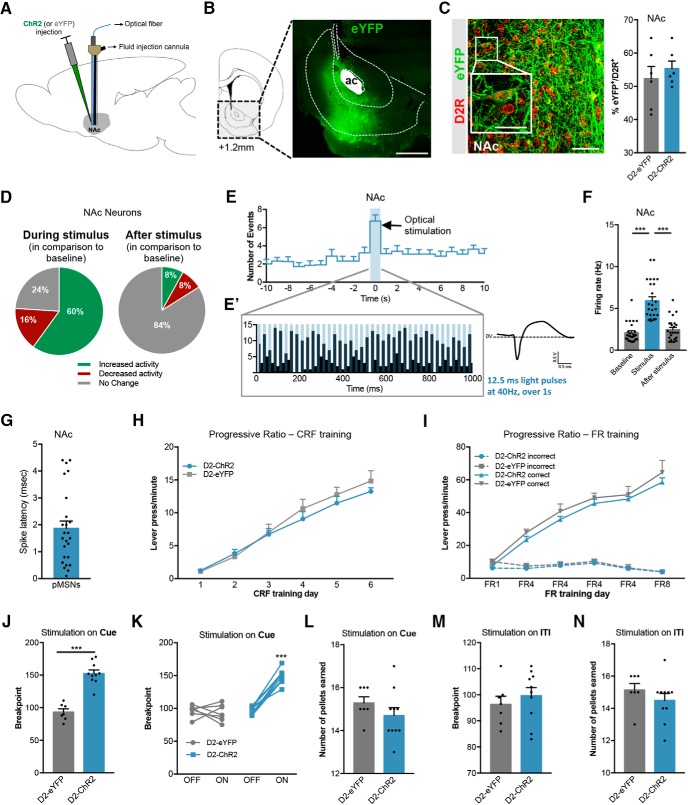
Optical stimulation of NAc D2-MSNs increases motivation. ***A***, AAV5-D2-ChR2(H134R)-eYFP (D2-ChR2 group) or AAV5-D2-eYFP (D2-eYFP group) was unilaterally injected in the NAc of Wistar han rats. A hybrid cannula was placed in the NAc to allow simultaneous delivery of fluids and optical stimulation. ***B***, Expression of eYFP was confirmed by YFP immunostaining. Scale bar: 500 μm; numbers represent distance to bregma in millimeters. ***C***, Representative immunostaining for D2R and eYFP in the NAc of an animal injected with AAV5-D2-ChR2(H134R)-eYFP. Scale bar: 100 μm, inset scale bar: 50 μm. More than 50% of D2-MSNs were transfected (*n* = 6 animals/group). ***D***, On D2-MSN optical stimulation (12.5-ms light pulses at 40 Hz, during 1 s), 60% of cells increased activity, 16% decreased, and 24% did not respond in comparison with baseline (*n* = 25 cells from four rats). ***E***, Time histogram of NAc electrophysiological single units in response to optical stimulus (average of 25 cells; blue stripe corresponds to laser stimulation). ***E’***, Example of a ChR2 neuron that responds to each pulse of stimulation. Right, Example of a representative MSNs wave form. ***F***, Increase in NAc average firing rate during optogenetic stimulation of D2-MSNs. ***G***, Spike latency in response to D2-MSN optical stimulation. ***H***, CRF training sessions of the PR test. ***I***, FR training sessions of the PR test. ***J***, Optogenetic activation of D2-MSNs during cue exposure strongly enhanced breakpoint. ***K***, All animals increase breakpoint in the session with D2-MSN stimulation (ON versus OFF session). ***L***, Number of pellets consumed in the PR session with stimulation was similar between groups. ***M***, Optogenetic activation of D2-MSNs during ITI does not alter breakpoint. ***N***, Number of pellets earned in the PR session with stimulation on ITI was similar between groups. n_D2-eYFP_ = 7; n_D2-ChR2_ = 10. Error bars denote SEM; ***p < 0.001 (Extended Data Figs. 1-1, 1-2).

To specifically modulate the activity of NAc D2R-expressing neurons, we injected in the NAc of rats a construct containing channelrhodopsin (ChR2) in fusion with enhanced yellow fluorescent protein (eYFP) under the control of the D2R minimal promoter (pAAV-D2Rp-hChR2(H134R)-eYFP), or the control eYFP virus (pAAV-D2Rp-eYFP; Fig. 1*A*,*B*; Extended Data Fig. 1-2; Soares-Cunha et al., 2016a; Zalocusky et al., 2016). Nearly 60% of NAc D2R-expressing neurons were successfully transfected with ChR2 or eYFP (D2R^+^/eYFP^+^ cells; Fig. 1*C*). In addition, only 1.5% of eYFP^+^ cells were D1R^+^; and 2% were ChAT^+^. Forty % of ChAT^+^ cells (CINs) were transfected since they express eYFP (Extended Data Fig. 1-2).

10.1523/ENEURO.0386-18.2018.f1-1 Extended Data Figure 1-1 Experimental design. ***A***, Animals from Group I were subjected to stereotaxic surgeries for injection of D2-ChR2 or D2-eYFP and optic fiber placement in the NAc, and let to recover from surgery for two weeks; after recovering, animals performed the PR task. On the PR session day, animals were killed 90 min after the beginning of the session for c-fos analysis and IF analysis. ***B***, Animals from Group II were subjected to the same protocol as Group I; one week after performing behavior in naïve conditions, animals were injected in the NAc on 1 d with the drug and on the other day with vehicle (counterbalanced within groups for treatment between the two test days) before PR performance. This test was repeated for all drugs with one week of interval between treatments. ***C***, Animals from Group III were subjected to stereotaxic surgeries for injection of D2-ChR2 or D2-eYFP in the NAc and optic fiber placement in the VP and performed the PR task as above. ***D***, Animals from Group IV were subjected to the same NAc surgery and were used for *in vivo* single-cell electrophysiological recordings in the NAc, VP, and VTA. Download Figure 1-1, TIF file.

**Figure 2.**
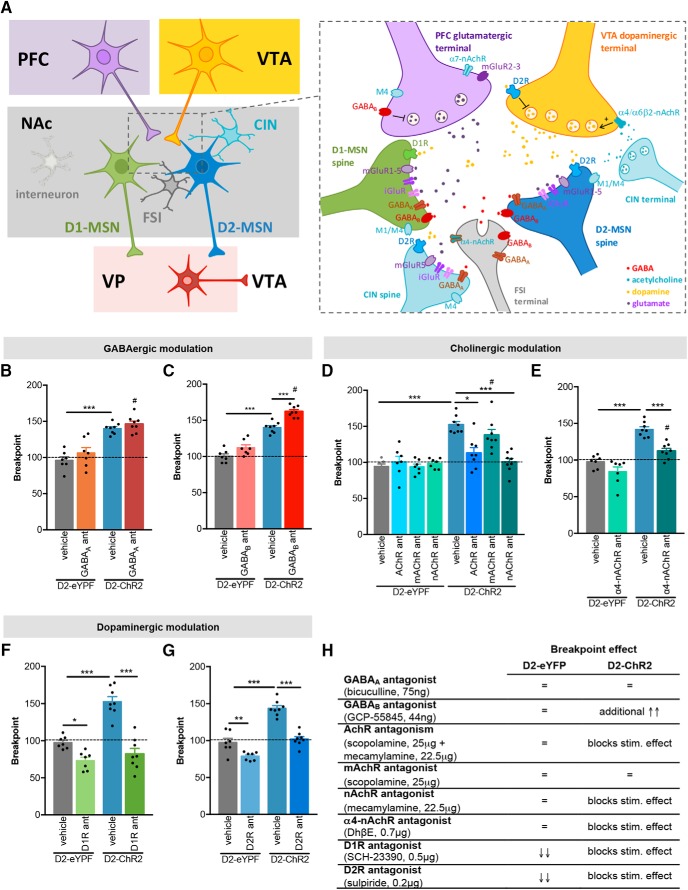
Effects of different antagonists in motivation. ***A***, Simplified schematic representation of NAc microcircuit. Left, The NAc receives cortical (prefrontal cortex (PFC)) glutamatergic inputs and VTA dopaminergic inputs. NAc D1- and D2-MSNs send GABAergic projections to VP, which in turn projects back to the NAc (not represented) and to VTA (among other regions). Besides MSNs, the NAc contains CINs and GABAergic interneurons of different natures, including FSIs, which tightly regulate striatal activity. Right, Expression of different neurotransmitter receptors in striatal neurons and terminals. Of relevance to mention that CINs also express dopamine receptor D2R and can stimulate dopamine release from VTA terminals mainly in a α4β2*nAchR- or α6β2*nAchR-dependent manner. Activation of D2R autoreceptors located in VTA terminals also controls dopamine release. iGluR: ionotropic glutamate receptors; mGluR: metabotropic glutamate receptors; nAchR: nicotinic (ionotropic) cholinergic receptors; M1/M4: muscarinic (metabotropic) cholinergic receptors. ***B–G***, Effects of different receptor antagonists in behavior. Rats were injected in the NAc with a specific antagonist immediately before the PR test with D2-MSNs optogenetic activation. ***B***, GABA_A_ receptor antagonist did not alter breakpoint of control D2-eYFP animals, nor of D2-ChR2-stimulated animals. ***C***, GABA_B_ receptor antagonist did not alter breakpoint of control animals, but it further increased the breakpoint of D2-ChR2-stimulated animals. ***D***, Injection of mAChR + nAChR antagonist combination abolished the increased breakpoint of D2-ChR2-stimulated animals. This effect is mediated mainly by nAChR since mecamylamine per se normalized breakpoint. ***E***, Local administration of α4-nAChR antagonist blocked the effect of D2-MSNs optogenetic activation. ***F***, D1R antagonist decreases the breakpoint of control D2-eYFP animals. In addition, the breakpoint enhancement induced by optogenetic activation of D2-MSNs was completely abolished by this treatment. ***G***, D2R antagonist originated a similar effect as D1R antagonist. ***H***, Summary of the effects of different antagonists in the breakpoint of stimulated D2-eYFP and D2-ChR2 animals (n_D2-eYFP_ = 7; n_D2-ChR2_ = 8). Error bars denote SEM; *p < 0.05, **p < 0.01, ***p < 0.001, #*p* < 0.001 and refers to the comparison D2-eYFP treated versus D2-ChR2 treated (Extended Data Figs. 2-1, 2-2).

10.1523/ENEURO.0386-18.2018.f1-2 Extended Data Figure 1-2 Confirmation of optic fiber location and expression specificity of Group I. ***A***, Optic fiber placement for D2-eYFP (grey) and D2-ChR2 (blue) rats (n_D2-eYFP_ = 7; n_D2-ChR2_ = 10). ***B***, Number of D2R^+^ and eYFP^+^ cells per area as evaluated by IF. Almost all of eYFP^+^ cells are also D2R^+^, confirming the specificity of the construct. ***C***, Number of D1R^+^ and eYFP^+^ cells per area. ***D***, Number of ChAT^+^ and eYFP^+^ cells. Only a few D1R^+^ and ChAT^+^ cells express the construct (n_D2-eYFP_ = 6; n_D2-ChR2_ = 6). Error bars denote SEM. Download Figure 1-2, TIF file.

Using single-cell *in vivo* electrophysiology, we showed that D2-MSN optical stimulation (40 Hz, 40 light pulses at 12.5 ms) significantly increases NAc firing rate during stimulation in comparison with baseline, and 84% of the cells return to basal activity after stimulation (*F*_(2,48)_ = 76.7, *p* < 0.000, one-way ANOVA; Fig. 1*D–F*). A total of 68% of recorded cells increased activity, 16% decrease, and 24% did not change activity in response to stimulation. Spike latency was ∼2 ms (Fig. 1*G*).

After, animals were submitted to PR test (Extended Data Fig. 1-1) to evaluate their willingness to work for a food reward, a direct measure of individual motivation. During CRF training, both groups increased lever pressing throughout days in a similar manner (*F*_(1,15)_ = 0.43, *p* = 0.522, two-way ANOVA; Fig. 1*H*). Likewise, all animals increased lever pressing in the FR schedule days in the active versus nonactive lever (*F*_(3,30)_ = 126.8, *p* < 0.000, two-way ANOVA; Fig. 1*I*).

In agreement with previous findings (Soares-Cunha et al., 2016a), D2-MSNs optical stimulation (40 light pulses of 12.5 ms at 40 Hz) occurring at the same time as the conditioned stimulus (light above the active lever), induced a significant increase in the breakpoint of D2-ChR2 rats in comparison with D2-eYFP-stimulated rats (63.6% increase; *t*_(15)_ = 7.7, *p <* 0.000, unpaired *t* test; Fig. 1*J*). All D2-ChR2 rats displayed a significant increase in the breakpoint in the session with optical stimulation (ON) in comparison with the session without stimulation (OFF; two-way ANOVA *post hoc*, *p <* 0.000; Fig. 1*K*). This increase in motivation was not due to differences in the number of food pellets earned during the PR session (*t*_(15)_ = 1.5, *p* = 0.1380, unpaired *t* test; Fig. 1*L*). Stimulation occurring during the ITI had no effect on motivation (Fig. 1*M*,*N*), proving that the positive effect of stimulation in behavior was restricted to particular stages of the test.

### Increase in motivation is dependent on NAc GABA signaling

MSNs are GABAergic in nature and synapse within each other in the NAc (Dobbs et al., 2016). Besides, local interneurons provide an additional source of GABA that also controls MSNs activity (Fig. 2*A*; Tepper et al., 2004).

10.1523/ENEURO.0386-18.2018.f2-1 Extended Data Figure 2-1 Representative image of viral infection extent and cannula entry site (numbers represent distance to bregma; scale bar: 1 mm); optic fiber placement for D2-eYFP (grey) and D2-ChR2 (blue) rats of Group II (n_D2-eYFP_ = 7; n_D2-ChR2_ = 8). Download Figure 2-1, TIF file.

10.1523/ENEURO.0386-18.2018.f2-2 Extended Data Figure 2-2 Number of pellets consumed during the PR session with optical stimulation with previous administration of different antagonists. ***A***, GABA_A_ receptor antagonist (bicuculline, 75 ng). ***B***, GABA_B_ receptor antagonist (GCP-55845, 44 ng). ***C***, mAChR antagonist (scopolamine, 25 μg) + nAChR antagonist (mecamylamine, 22.5 μg). ***D***, α4-nAChR antagonist (DHβE, 0.7 μg). ***E***, D1R antagonist (SCH-23390, 0.25 μg). ***F***, D2R antagonist (sulpiride, 0.2 μg). Error bars denote SEM; *p < 0.05. Download Figure 2-2, TIF file.

To further understand the impact of GABAergic neurotransmission in the control of D2-MSNs-mediated enhancement of motivation, we used hybrid cannulas, which allow dual delivery of drugs and light in the same region (Extended Data Figs. 1-1, 2-1). Immediately before behavioral testing and optogenetic activation of D2-MSNs, we injected in the NAc either a GABA_A_ receptor antagonist (bicuculline, 75 ng) or a GABA_B_ receptor antagonist (CGP 55845 hydrochloride, 44 ng), in dosages that have been shown previously to induce a behavioral effect (Giorgetti et al., 2002; Kandov et al., 2006; Ikeda et al., 2010).

For GABA_A_ receptor antagonist, we found no significant effect of treatment but there was a group effect, with D2-ChR2-stimulated animals presenting increased breakpoint (two-way ANOVA; treatment effect: *F*_(1,13)_ = 0.1, *p* = 0.117; group effect: *F*_(1,13)_ = 118.8, *p <* 0.000; Fig. 2*B*). For GABA_B_ receptor antagonist, there was a significant effect of treatment and group (two-way ANOVA; treatment effect: *F*_(1,13)_ = 30.7, *p <* 0.000; group effect: *F*_(1,13)_ = 193, *p <* 0.000; Fig. 2*C*).

None of the GABA antagonists alters the breakpoint of control D2-eYFP animals (Fig. 2*B*,*C*), although there was a trend for increased number of lever presses with GABA_B_ receptor antagonist treatment (12% increase; *p* = 0.070, two-way ANOVA *post hoc*). GABA_A_ receptor antagonist administration before D2-MSNs stimulation did not impair the breakpoint enhancement (D2-ChR2 vehicle vs D2-ChR2 GABA_A_ antag, *p* = 0.787, two-way ANOVA *post hoc*; Fig. 2*B*). However, administration of GABA_B_ receptor antagonist led to an additional increase in the breakpoint of D2-stimulated animals (15.8% increase; *p* < 0.000, two-way ANOVA *post hoc*; Fig. 2*C*). No differences were found between groups in the number of pellets earned during the session (Extended Data Fig. 2-2).

These results suggest that GABA signaling arising from MSNs or local interneurons can modulate motivational drive in a GABA_B_-dependent manner.

### Increase in motivation is dependent on NAc cholinergic signaling

In addition to GABAergic modulation, MSNs activity is tightly controlled by CINs (Fig. 2*A*), which are able to control dopamine release from VTA terminals in the NAc (Cachope et al., 2012), promoting behavioral conditioning (Witten et al., 2010).

Using a similar approach as above, we injected in the NAc a combination of mAChR and nicotinic AChR (nAChR) antagonists before PR paradigm (scopolamine, 25 μg; mecamylamine, 22.5 μg, respectively; dosages previously validated; Nadal et al., 2002; Rahman and McBride, 2002; Yee et al., 2011; Perry et al., 2014). Treatment had a significant effect on behavior (*F*_(3,39)_ = 6.3, *p* = 0.001, two-way ANOVA; Fig. 2*D*). Blockade of cholinergic signaling significantly abolished the motivation enhancement induced by optogenetic D2-MSN activation (D2-ChR2 vehicle vs D2-ChR2 mAChR + nAChR antag, *p* < 0.000, two-way ANOVA *post hoc*; Fig. 2*D*).

Further studies using either one of the antagonists revealed that this blockage was mediated by nAChR (D2-ChR2 vehicle vs D2-ChR2 nAChR antag, two-way ANOVA *post hoc*, *p* < 0.000; Fig. 2*D*). No differences in the number of pellets earned during the session were found (Extended Data Fig. 2-2).

In the NAc, MSNs express mAChR (M1 and M4; Yan et al., 2001) but not nAChR (Jones et al., 2001; Jones and Wonnacott, 2004). The later receptors are mainly expressed in VTA dopaminergic terminals (Hill et al., 1993) and some GABAergic interneurons (Koós and Tepper, 1999; Fig. 2*A*). Tonic striatal ACh is able to promote dopamine release through β2-subunit-containing (β2*)-nAChR receptors in VTA terminals (Rice and Cragg, 2004). Using different KO strains, Champtiaux and colleagues proposed that a combination of α6β2* and α4β2^*^ nAChRs mediate the endogenous cholinergic modulation of dopamine release at the terminal level (Champtiaux et al., 2003). Considering this, we injected DHβE (0.7 μg; dosage validated; Löf et al., 2007), an antagonist of α4 subunit of nAChR, in the NAc before performing the PR test. By blocking α4 receptors, we are abolishing at least 50% of dopamine release in the NAc (Champtiaux et al., 2003).

Treatment using α4 antagonist had a significant effect on behavioral performance (*F*_(1,13)_ = 43.0, *p* < 0.000, two-way ANOVA; Fig. 2*E*). No effect in the breakpoint of control animals was found, yet, this treatment abolished the enhancement of breakpoint induced by D2-MSN stimulation (20.8% decrease; *p* < 0.000, two-way ANOVA *post hoc*). No effect on the number of pellets earned during the session was found (Extended Data Fig. 2-2).

These results suggest that cholinergic activation of VTA terminals is required for the observed behavioral effect of D2-MSN stimulation.

### Enhancement of motivation by D2-MSN activation requires dopamine signaling through D1R and D2R

Activating α6β2^*^ and/or α4β2^*^ nAChRs in VTA terminals greatly enhances dopamine release in the NAc (Wonnacott et al., 2000; Cachope et al., 2012), and our previous results suggested that cholinergic modulation of VTA terminals was necessary for the observed motivation enhancement induced by D2-MSN optogenetic activation. Thus, we next tried to clarify the role of NAc dopamine receptors D1R and D2R in this process. To do so, we injected in the NAc before performance of PR test with optogenetic stimulation of D2-MSNs, R(+)-SCH-23390 hydrochloride (0.5 μg; D1R antagonist) or sulpiride (0.2 μg; D2R antagonist) in doses that were previously shown to have a behavioral effect (Vezina et al., 1994).

Both D1R and D2R antagonist treatment had a significant effect (two-way ANOVA; D1R antag: *F*_(1,13)_ = 65.7, *p* < 0.000; D2R antag: *F*_(1,13)_ = 56.8, *p* < 0.000; Fig. 2*F*,*G*). Interestingly, both antagonists caused a reduction in the breakpoint of control D2-eYFP animals (D1R antagonist: 25.1% decrease, *p* = 0.047, two-way ANOVA *post hoc*; D2R antagonist: 26.2% decrease, *p* = 0.013, two-way ANOVA *post hoc*).

Additionally, pharmacological inhibition of either D1R or D2R abolished the increase in motivation induced by D2-MSN optogenetic activation (D2-ChR2 vehicle vs D2-ChR2 D1R antag: *p* < 0.000, two-way ANOVA *post hoc*; D2-ChR2 vehicle vs D2-ChR2 D2R antag: *p* < 0.000, two-way ANOVA *post hoc*). A reduction in the number of pellets consumed in D1R-treated D2-eYFP rats was found (*p* = 0.0164, two-way ANOVA *post hoc*; Extended Data Fig. 2-2). No significant differences in the number of pellets consumed were found in other groups.

These results suggest that the motivation improvement is dependent on both types of dopamine receptor signaling in the NAc.

### Optogenetic stimulation of NAc D2-MSNs recruits the VP and the VTA

The preceding results suggested a dopamine-dependent effect of D2-MSN optogenetic activation in motivation (summarized in Fig. 2*H*). D2-MSNs do not directly project to VTA but indirectly modulate VTA dopaminergic activity through the VP (Wu et al., 1996; Floresco et al., 2003; Grace et al., 2007; Hjelmstad et al., 2013; Kupchik et al., 2015). So, we next examined the pattern of expression of c-fos, an immediate early gene used as a marker of neuronal recruitment, after the PR test in the NAc and connected regions.

**Figure 3.**
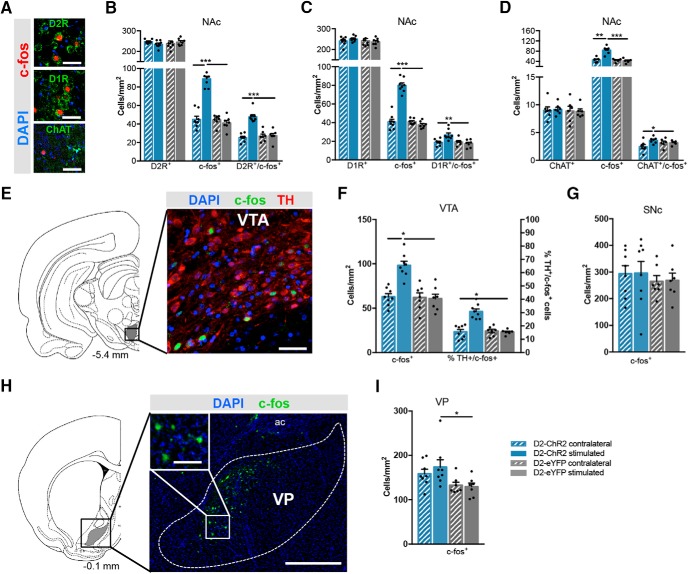
Effect of optogenetic activation of D2-MSNs in the NAc and downstream targets. ***A***, Representative immunostaining c-fos and D2R, D1R, or ChAT in the NAc. Scale bar: 80 μm (n_D2-ChR2_ = 8; n_D2-eYFP_ = 7). ***B***, Counting of D2R^+^ and c-fos^+^ cells in the NAc. D2-MSNs stimulation recruits more D2^+^ neurons in comparison to nonstimulated side (contralateral). Stimulated D2-ChR2 animals present increased number of D2^+^/c-fos^+^ neurons in comparison to stimulated D2-eYFP animals (control group). ***C***, Counting of D1R^+^ and c-fos^+^ cells in the NAc, showing an increase in D1^+^/c-fos^+^ in stimulated versus contralateral side (or vs D2-eYFP-stimulated animals). ***D***, Counting of ChAT^+^ and c-fos^+^ cells in the NAc, showing an increase in ChAT^+^/c-fos^+^ in stimulated versus contralateral side (or vs D2-eYFP-stimulated animals. ***E***, Representative immunostaining for TH and c-fos in the VTA (scale bar: 100 μm). ***F***, Respective quantification of positive cells. D2-MSN stimulation increases the number of TH^+^ neurons in the VTA. ***G***, SN c-fos^+^ cells counting showing no effect of stimulation. ***H***, Representative immunostaining for c-fos in the VP (scale bar: 500 μm; scale bar in inset: 100 μm). ***I***, Stimulated D2-ChR2 animals present increased c-fos staining in the VP comparison to control D2-eYFP animals; interestingly, no significant differences were found between stimulated versus nonstimulated side. Error bars denote SEM; *p < 0.05, **p < 0.01, ***p < 0.001 (Extended Data Fig. 3-1).

Stimulated D2-ChR2 rats showed a significant increase in c-fos staining in NAc D2R-expressing neurons, when compared with stimulated control D2-eYFP rats (*t*_(13)_ = 12.0, *p* < 0.000, unpaired *t* test; Fig. 3*A*,*B*; Extended Data Fig. 3-1), and when compared with the nonstimulated side (*t*_(7)_ = 7.4, *p* = 0.0002, paired *t* test). This increase in c-fos expression was also observed in NAc D1R-expressing neurons when comparing D2-ChR2 with D2-eYFP rats (*t*_(13)_ = 3.7, *p* = 0.0028, unpaired *t* test; Fig. 3*A*,*C*), and with the contralateral nonstimulated side (*t*_(7)_ = 5.3, *p* = 0.0011, paired *t* test).

10.1523/ENEURO.0386-18.2018.f3-1 Extended Data Figure 3-1 IF against GFP and D1R or D2R in D2-EGFP reporter strain. ***A***, Representative image of a section of a D2-GFP animal labelled with anti-GFP and anti-dopamine receptor D2 (scale bar: 50 μm). ***B***, Representative image of a section of a D2-GFP animal labelled with anti-GFP and anti-D1R (scale bar: 50 μm). ***C***, Respective quantification of IF; 54.4% of total cells were GFP^+^, in agreement with half of the NAc cells being D2-MSNs. Of those GFP^+^ cells, 83% were D2R^+^ and 17% D2R^-^; whereas most (73%) of these cells were D1R^-^. Error bars denote SEM. Download Figure 3-1, TIF file.

ChAT-expressing neurons also presented increased c-fos expression when comparing D2-ChR2 with D2-eYFP rats (*t*_(13)_ = 5.7, *p* < 0.000, unpaired *t* test; Fig. 3*A*,*D*), or comparing with contralateral nonstimulated side (*t*_(7)_ = 4.0, *p* = 0.0053, paired *t* test).

In addition, we evaluated the number of c-fos^+^ cells in accumbal downstream regions: the VTA, which is innervated solely by NAc D1-MSNs (Bocklisch et al., 2013); the VP, which is directly innervated by NAc D1- and D2-MSNs (Creed et al., 2016); and the substantia nigra pars compacta (SNc) as a control region, since it is mainly innervated by dorsal striatum MSNs (Gerfen, 1984).

A significant increase in VTA c-fos^+^ cells was observed in D2-ChR2 rats in comparison to D2-eYFP-stimulated rats (*t*_(13)_ = 5.3, *p* < 0.000, unpaired *t* test; Fig. 3*E*,*F*), or when comparing with contralateral side (*t*_(7)_ = 4.6, *p* = 0.0024, paired *t* test); from these, around 30% were dopaminergic neurons (*t*_(13)_ = 7.1, *p* < 0.000, unpaired *t* test). A similar increase in c-fos was observed in the VP of D2-ChR2 in comparison with D2-eYFP rats (*t*_(13)_ = 2.3, *p* = 0.039, unpaired *t* test; Fig. 3*H*,*I*). However, no significant difference in c-fos was found between stimulated and contralateral VP in D2-ChR2 rats (*t*_(7)_ = 1.2, *p* = 0.258, paired *t* test). D2-MSNs accumbal stimulation did not alter c-fos expression in the SN (Fig. 3*G*).

### Optogenetic activation of NAc-VP terminals recapitulates motivation enhancement

**Figure 4.**
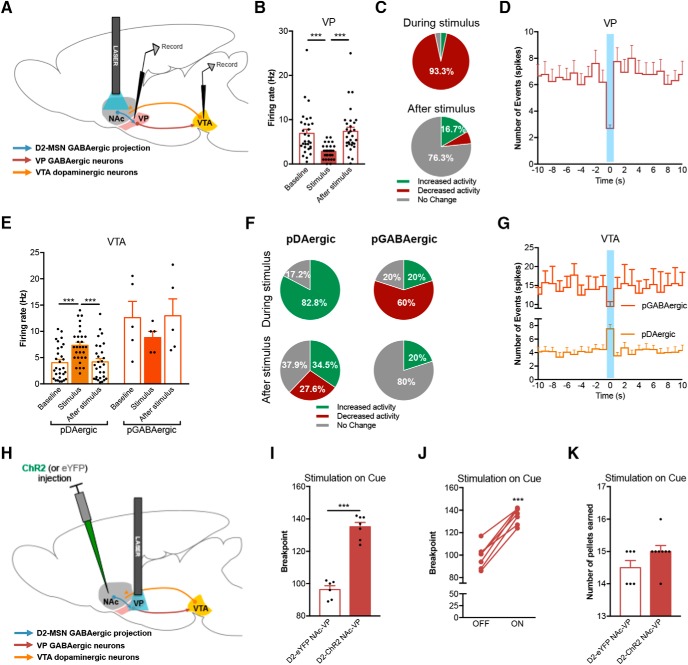
Activation of D2-MSN terminals in the VP increases motivation. ***A***, Schematic representation of the *in vivo* single-cell electrophysiological recordings with optogenetic manipulation of NAc D2-MSNs cell bodies. ***B***, NAc D2-MSN optical stimulation (40 Hz, 12.5-ms pulses for 1 s) decrease net firing rate of VP neurons. ***C***, 93.3% of VP cells decrease firing rate and 6.7% did not respond to stimulation (*n* = 30 cells/four rats). ***D***, Time histogram showing the number of events in the VP before, during, and after a 40-Hz stimulus of NAc D2-MSNs. ***E***, D2-MSN optical stimulation increase the net firing rate of pDAergic neurons of the VTA, with no significant changes in the net firing rate of pGABAergic neurons (n_pDAergic_ = 29 cells/four rats; n_GABAergic_ = 5 cells/four rats). ***F***, 82.8% of pDAergic cells increased firing rate in response to stimulation; most of cells returned to baseline activity after the stimulus. pGABAergic neurons presented a majority of inhibitory responses to D2-MSN stimulation. ***G***, Time histogram showing the number of events in the VTA before, during, and after a 40-Hz stimulus of NAc D2-MSNs. ***H***, Strategy used for optogenetic stimulation of D2-MSN terminals in the VP (D2-ChR2 NAc-VP group). ***I***, Optogenetic activation of D2-MSN-VP terminals during cue exposure strongly enhanced breakpoint. ***J***, All animals increase breakpoint in the session with stimulation (ON) versus nonstimulation session (OFF). ***K***, Number of pellets consumed in the PR session was similar between groups. (n_D2-eYFP NAc-VP_ = 6; n_D2-ChR2 NAc-VP_ = 8). Error bars denote SEM; ***p < 0.001 (Extended Data Fig. 4-1).

Next, we analyzed the activity of the VP and VTA during D2-MSN optogenetic stimulation using *in vivo* single-cell electrophysiology (Fig. 4*A*).

10.1523/ENEURO.0386-18.2018.f4-1 Extended Data Figure 4-1 Additional data from optogenetic activation experiments. ***A***, Spike latency in the VP and VTA neurons in response to NAc D2-MSN optogenetic stimulation. VP neurons present reduced spike latency to fire, consistent with a monosynaptic input from D2-MSNs, whereas VTA neurons present spike latencies indicative of polysynaptic modulation. ***B***,***C***, CRF and FR learning curves of D2-eYFP and D2-ChR2 NAc-VP animals. Download Figure 4-1, TIF file.

Concordant with a GABAergic input, NAc D2-MSN stimulation elicited an overall reduction in the firing rate of the VP (*F*_(2,87)_ = 10.6, *p* < 0.000, one-way ANOVA; Fig. 4*B*), with an average spike latency of 5.7 ms (Extended Data Fig. 4-1*A*), consistent with the expected monosynaptic input from the NAc to VP. More than 90% of recorded neurons in the VP decreased their activity during stimulation, which normalized thereafter (Fig. 4*C*,*D*).

Conversely, in the VTA, we found a significant increase in global firing rate of putative VTA dopaminergic neurons (pDAergic; *F*_(2,56)_ = 17.6, *p* < 0.000, one-way ANOVA; Fig. 4*E*), with an average spike latency of 170 ms (Extended Data Fig. 4-1*A*), indicative of polysynaptic modulation. Of these pDAergic neurons, 82.8% increased activity during stimulation (Fig. 4*F*,*G*). No significant differences were observed in the activity of pGABAergic VTA neurons, although there was a trend for decreased activity during D2-MSNs stimulation (Fig. 4*E*,*G*).

The previous data suggested an indirect modulation of VTA activity through the VP, so we decided to optogenetically stimulate D2-MSN terminals in the VP during the PR test (Fig. 4*H–K*). Regarding training, both groups learned in a similar manner [CRF: *F*_(1,72)_ = 0.0, *p* = 0.856, two-way ANOVA (Extended Data Fig. 4-1*B*); FR: *F*_(3,24)_ = 180.4, *p* < 0.000, two-way ANOVA (Extended Data Fig. 4-1*C*)].

Optical stimulation (40 light pulses of 12.5 ms at 40 Hz) of D2-MSN-VP terminals elicited a significant increase in the breakpoint of ChR2-stimulated rats in comparison with control-stimulated rats (40% increase; *t*_(11)_ = 10.7, *p* < 0.000, unpaired *t* test; Fig. 4*I*). All D2-ChR2 NAc-VP rats displayed a significant increase in breakpoint in the session with optical stimulation (ON) in comparison with the OFF session (*t*_(6)_ = 10.2, *p* < 0.000, paired *t* test; Fig. 4*J*). No differences in the number of food pellets earned during the PR session were found (*t*_(12)_ = 1.7, *p* = 0.112, unpaired *t* test; Fig. 4*K*).

## Discussion

**Figure 5.**
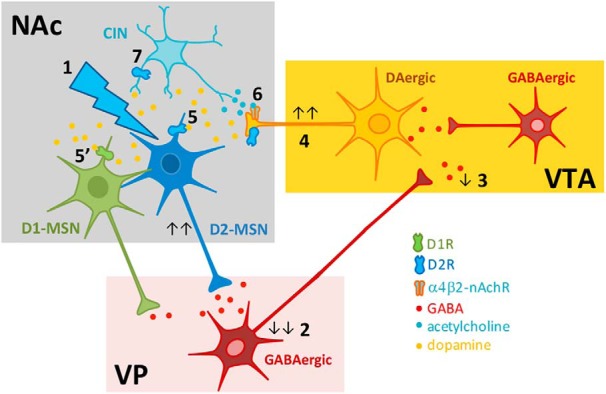
Proposed model for D2-MSNs optogenetic activation effects in NAc microcircuit. NAc D2-MSNs send GABAergic projections to VP neurons, which in turn provide a tonic inhibitory input to the VTA (**1**). Optogenetic activation of D2-MSNs reduces VP net activity (**2**), reducing VP-to-VTA inhibitory tone (**3**). This triggers an increase in VTA dopaminergic activity (**4)**. These VTA dopaminergic signals require D1R and D2R signaling in the NAc (5’, **5**). Interestingly, cholinergic-dependent control of VTA dopaminergic terminals in the NAc (via α4-nAChR) is essential for this process (**6**). (**7**) Optical stimulation can also be activating D2-expressing CINs that strongly influence dopamine release and shape behavior.

Local microcircuits in combination with excitatory and inhibitory inputs from upstream regions play an important role in striatal function. Here, we show that activation of D2-MSNs during cue exposure increases willingness to work in the PR test, and that a concerted action of different neurotransmitter systems in the striatum is required for this behavioral effect (Fig. 5).

We first evaluated the impact of GABAergic transmission since GABAergic MSNs highly synapse within each other in the NAc (Sesack and Pickel, 1990; Dobbs et al., 2016), providing a weak lateral inhibitory network (feedback inhibition; Tepper et al., 2008). This MSN-MSN reciprocal regulation mainly occurs in a GABA_A_ receptor mediated manner (Tunstall et al., 2002). Our results suggest that the D2-MSN-driven enhancement in motivation is not dependent on GABAergic signaling, since neither GABA_A_ nor GABA_B_ antagonists normalized the phenotype. However, we do observe an additional increase in the breakpoint of both control and D2-MSN-stimulated animals on GABA_B_ antagonist administration in the NAc. Such finding is likely to rely on enhanced corticostriatal glutamatergic release on the blockade of presynaptic GABA_B_ receptors. In fact, MSNs express GABA_B_ receptors, application of exogenous GABA_B_ agonists does not lead to any MSN electrophysiological effect (Logie et al., 2013), although it significantly supresses glutamatergic inputs onto MSNs via a pre-synaptic mechanism (Nisenbaum et al., 1993; Logie et al., 2013). Apart from classic studies showing that NAc cue-evoked firing is abolished by VTA inactivation (Yun et al., 2004), there is also evidence that cue-evoked excitations of NAc core neurons depend on mPFC glutamatergic projections, and contribute to the behavioral response to reward-predictive cues (Ishikawa et al., 2008).

Yet, it is important to refer that although sparse, GABAergic interneurons (which do not express D2R; Tritsch and Sabatini, 2012) display highly branched dendritic and extensive axonal arborisations (Kawaguchi, 1997; Ibáñez-Sandoval et al., 2011; English et al., 2012) and are capable of exerting a powerful control over striatal excitability (feed-forward inhibition; Tepper et al., 2004, 2008). They also express GABA_B_ receptors (Logie et al., 2013), so the blockage of this specific feed-forward inhibition might also contribute for the observed increase in motivational drive.

In addition to local GABA control, the striatum also contains CINs, which have both excitatory and inhibitory effects in striatal MSNs (Sullivan and Brake, 2003; Pakhotin and Bracci, 2007; Witten et al., 2010). In primates, CINs exhibit multiphasic responses to motivationally salient stimuli that mirror those of midbrain dopamine neurons, being important for reward-related learning (Kitabatake et al., 2003; Joshua et al., 2008; Witten et al., 2010; Cachope et al., 2012). Since 80% of CINs express D2R (Alcantara et al., 2003), one can argue that our optogenetic stimulation protocol directly activates these interneurons, enhancing ACh release in the striatum. In line with this, we found an increase in ChAT^+^/c-fos^+^ neurons in stimulated animals.

*In vivo* selective activation of CINs is sufficient to elicit dopamine release directly in the NAc and independently of the soma, by activation of nAChRs in VTA terminals (Cachope et al., 2012; Threlfell et al., 2012). It has been suggested that these nAChR act as dynamic detectors of ACh concentrations, enhancing the contrast between tonic and burst dopaminergic firing (Brunzell et al., 2010). In an elegant study using different KO strains, Champtiaux and colleagues proposed that a combination of α6β2* and α4β2* nAChRs mediate endogenous cholinergic modulation of dopamine release at the VTA terminal level (Champtiaux et al., 2003). Here, we show that α4 antagonist, DHβE, blocks D2-MSN-dependent increase in motivation, suggesting that ACh-mediated dopamine release from VTA terminals is crucial for the observed behavioral effect. It is important to refer that besides CINs, the NAc may also receive cholinergic inputs from the laterodorsal tegmentum (Dautan et al., 2014), although the function of these projections remains completely unknown.

In the NAc, α4 nAChRs subunits are expressed mainly in VTA dopaminergic terminals but also in some GABAergic FSIs. So, the observed dampening of motivation with α4 antagonist could also depend on these interneurons. However, our data does not support this because GABA receptor antagonists did not abolish the optogenetic-induced behavioral effect.

In addition to local cholinergic control, our data suggests an indirect effect in VTA dopaminergic activity through the VP. First, c-fos analysis revealed increased recruitment of both VP and VTA regions. VP data are somehow surprising considering the GABAergic nature of accumbal-VP monosynaptic projections (Root et al., 2010; Kupchik et al., 2015). Although most studies associate c-fos expression with increased neuronal activity, at least one study has shown that activating striatal MSNs increases c-fos in the VP (Page and Everitt, 1993). Yet, rather than directly associate D2-MSN activation with this increase in c-fos in the VP, we just aim to illustrate that the VP is being differently recruited in stimulated animals. In fact, animals were killed 90 min after the beginning of the PR test, so c-fos reactivity is a sum of all neuronal events that occur during the test, and do not reflect only the optogenetic activation period.

D2-MSN stimulation decreased VP firing rate, and indirectly increased VTA dopaminergic activity, with less effects in GABAergic VTA neurons, consistent with the preferential innervation of VTA dopaminergic neurons by VP inputs (Mahler et al., 2014). So, our hypothesis is that D2-MSNs reduce the tonic VP-VTA inhibitory input, contributing for enhanced dopaminergic activity, which is known to boost motivational drive (Peciña et al., 2003; Cagniard et al., 2006). In fact, it was shown that inhibition of NAc afferents to the VP or direct infusion of GABAergic agonists into the VP, selectively increased the population activity of dopamine neurons, rising NAc dopamine efflux (Floresco et al., 2003). In line with this, we showed that optogenetic activation of D2-MSN terminals in the VP was sufficient to increase motivation. These findings are in agreement with the emerging notion that the VP is crucial for reward and motivation toward natural rewards and drugs of abuse. In fact, different subregions of the VP mediate different aspects of rewarded behavior, from motivation/incentive salience to reward prediction and consumption (Smith et al., 2009; Root et al., 2015). Yet, it is important to refer that VP is not only a relay area for indirect NAc inputs, since VP neuron responses can occur at a shorter latency than cue-elicited responses in NAc neurons (Richard et al., 2016), and that VP firing rate reflects the strength of incentive motivation (Ahrens et al., 2016).

The increased dopaminergic signals arising from the VTA act mainly (not exclusively since some interneurons also express dopamine receptors) on MSNs either by activating D1R or D2R. Local administration of either D1R or D2R antagonists decreases motivation in control animals, and also abolished D2-MSN-induced positive effects in motivation, indicating a synergistic effect of both MSN populations. In this perspective, it is important to refer that blockade of D2R would be expected to enhance activity of D2-MSNs since D2Rs are coupled to inhibitory G-proteins (Beaulieu and Gainetdinov, 2011). Yet, one has to bear in mind that D2R antagonists can also act in D2 auto-receptors in VTA terminals, disinhibiting presynaptic control of dopamine release (Anzalone et al., 2012).

Interestingly, D2-MSN optogenetic activation during cue exposure also indirectly recruited D1-MSNs, as assessed by an increase in the number of D1^+^/c-fos^+^ cells in the NAc on stimulation. Considering the proposed role for D1R-expressing neurons in reinforcement (Lobo et al., 2010; Kravitz et al., 2012), this activation probably also contributes for the behavioral output.

In summary, we show that NAc D2-MSN optogenetic activation enhances motivation through enhanced VTA-driven dopaminergic signaling. The behavioral effect was dependent on both D1R and D2R signaling in the NAc, suggesting that a coordinated action between these two striatal populations is needed to increase motivational levels.

## Synthesis

Reviewing Editor: Carmen Sandi, Swiss Federal Institute of Technology Decisions are customarily a result of the Reviewing Editor and the peer reviewers coming together and discussing their recommendations until a consensus is reached. When revisions are invited, a fact-based synthesis statement explaining their decision and outlining what is needed to prepare a revision will be listed below. The following reviewer(s) agreed to reveal their identity: Alexander Smith

The role of D2-MSN projections to the Ventral Pallidum is of broad interest to the field, given the described roles of this pathway in aversion and avoidance learning. In this manuscript, Soares-Cunha and colleagues eloquently describe a striatal microcircuit through which acetylcholine release onto D2-MSNs enhances motivation in a progressive ratio task. They combine optogenetics with in-vivo electrophysiology in order to show that stimulating accumbens D2-MSNs indeed causes an increase in firing of VTA dopamine neurons, and also increases the number of Fos+ neurons in both the VP and VTA. Importantly, optogenetic stimulation of D2-MSN terminals in the VP was able to recapitulate this effect. Furthermore, use of selective alpha4 nicotinic antagonists was able to block the effect of optogenetic stimulation, indicating a role for cholinergic interneurons in this phenomenon. The finding that D2-MSNs projecting to the ventral pallidum are involved in regulating motivational drive for rewards is of broad interest to those who study reward and affective neuroscience. The extended data supplement the figures in the main text and strengthen the manuscript from its earlier versions.

The two reviewers and editor agree that this manuscript makes an important contribution to the field. We only have the following minor requests for correction:

1. Limitations in the discussion: it will be important to indicate that, regarding D2 antagonism infusion, blockade of D2 receptors on D2-MSNs themselves would be expected to enhance activity of the MSNs since D2Rs are coupled to inhibitory G-proteins.

2. Line 302 - the authors cite anatomical studies for inhibitory connections between MSNs in the NAc. Dobbs et al., Neuron 2016 may be a more appropriate reference to show the functional consequences of lateral inhibition between MSN subtypes, as they do in the discussion.

3. Lines 408 - 410: “The VTA which is innervated solely by NAc D1-MSNs”, [this sentence should be referenced, particularly in light of Bocklisch et al., Science 2013] the VP, which is directly innervated by NAc D1- and D2-MSNs [should be referenced, ie. Creed et al., Neuron 2016]; and the substantia nigra pars compacta as a control region, since it is mainly innervated by dorsal striatum MSNs [Kravitz et al., 2012 did not do anatomical studies that support this reference].

4. Figure 2A. The ‘NAc microcircuit’ is confusing as it is currently presented, and should be at least embedded in a a larger schematic of VTA/VP/NAc/Cortex. As it stands, the components are dis-embodied and very difficult to understand, there are some terminals, or are they spines, axons or cell bodies? What are these fractions of cells supposed to represent and to where to they project?
