# Erratum: Soares-Cunha et al., “Nucleus Accumbens Microcircuit Underlying D2-MSN-Driven Increase in Motivation”

**DOI:** 10.1523/ENEURO.0144-25.2025

**Published:** 2025-04-18

In the article “Nucleus Accumbens Microcircuit Underlying D2-MSN-Driven Increase in Motivation,” by Carina Soares-Cunha, Bárbara Coimbra, Ana Verónica Domingues, Nivaldo Vasconcelos, Nuno Sousa, and Ana João Rodrigues, which was published online on April 19, 2018, a typographical error was identified in the citation of an Extended Data figure. In the “Immunofluorescence (IF)” subsection of the Materials and Methods, the last sentence should cite Extended Data Fig. 3-1, not 5-1, reading, “Anti-D1R and anti-D2R antibodies were previously validated (Luedtke et al., 1999; Basu et al., 2004; Luessen et al., 2016; Extended Data Fig. 3-1).”

The authors apologize for the error.

