# Supplementary figures and images for: Nucleus Accumbens Microcircuit Underlying D2-MSN-Driven Increase in Motivation
Source: eNeuro. 2018 May 17;5(2):ENEURO.0386-18.2018. doi: 10.1523/ENEURO.0386-18.2018 (PMC5957524; doi:10.1523/ENEURO.0386-18.2018)

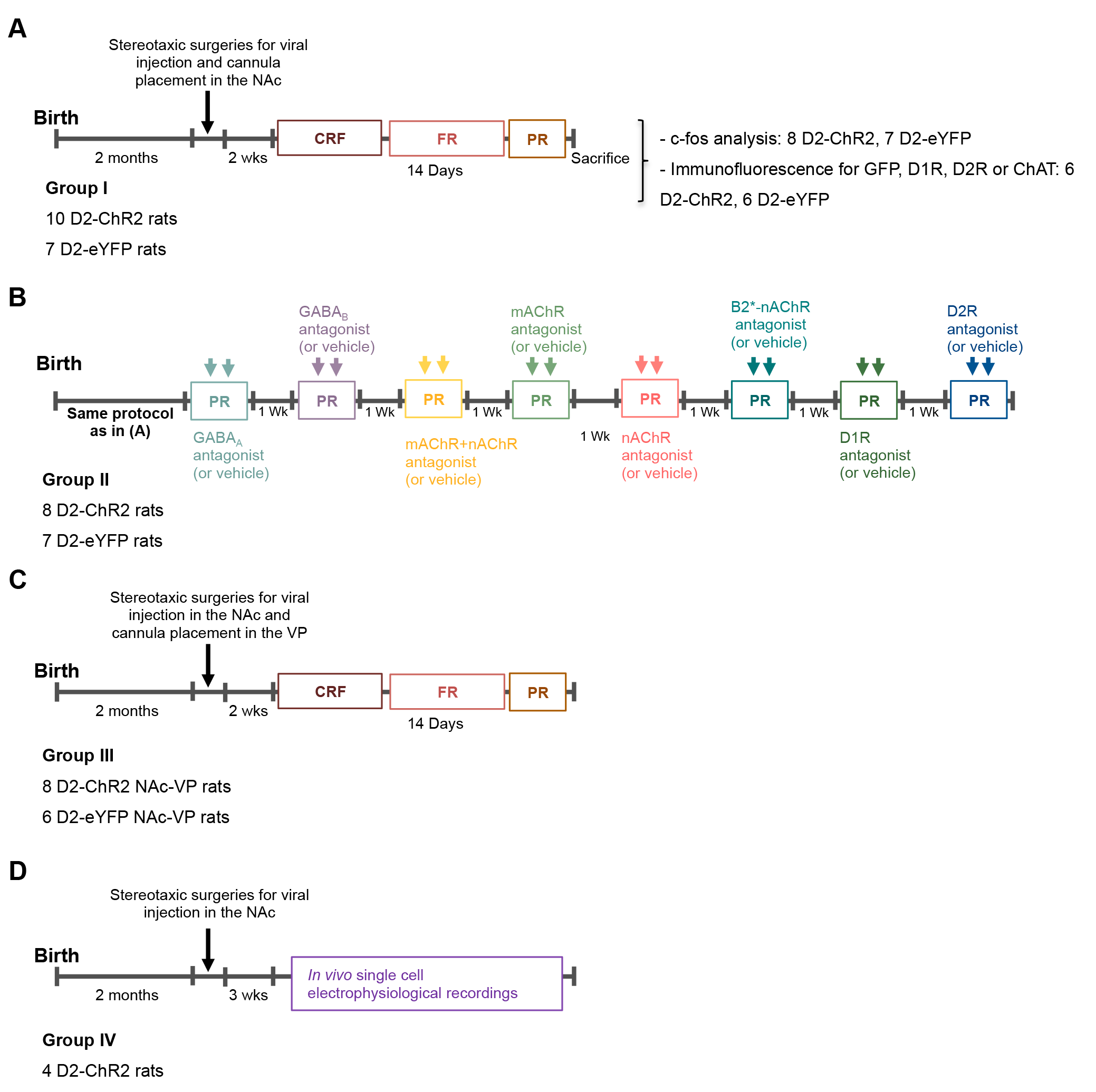

Supplement: Extended Data Figure 1-1 — Experimental design. A, Animals from Group I were subjected to stereotaxic surgeries for injection of D2-ChR2 or D2-eYFP and optic fiber placement in the NAc, and let to recover from surgery for two weeks; after recovering, animals performed the PR task. On the PR session day, animals were killed 90 min after the beginning of the session for c-fos analysis and IF analysis. B, Animals from Group II were subjected to the same protocol as Group I; one week after performing behavior in naïve conditions, animals were injected in the NAc on 1 d with the drug and on the other day with vehicle (counterbalanced within groups for treatment between the two test days) before PR performance. This test was repeated for all drugs with one week of interval between treatments. C, Animals from Group III were subjected to stereotaxic surgeries for injection of D2-ChR2 or D2-eYFP in the NAc and optic fiber placement in the VP and performed the PR task as above. D, Animals from Group IV were subjected to the same NAc surgery and were used for in vivo single-cell electrophysiological recordings in the NAc, VP, and VTA. Download Figure 1-1, TIF file. [file sup_enu-eN-NWR-0386-18-s02.tif]

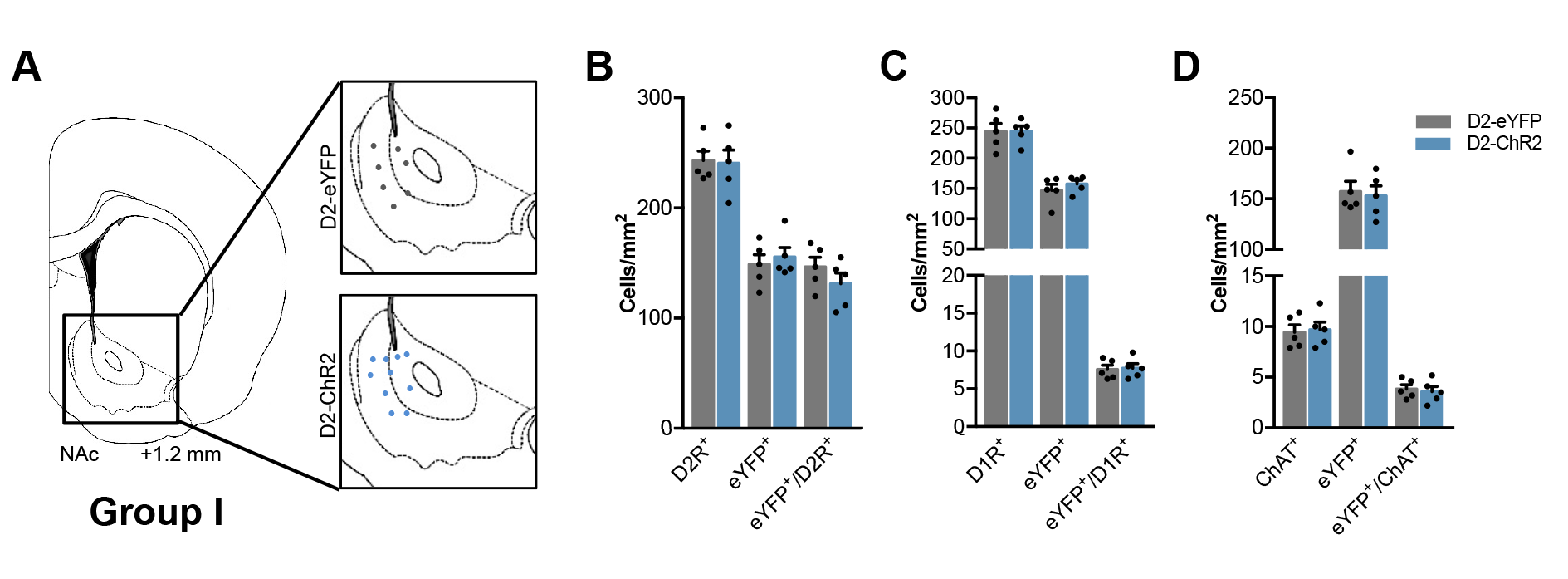

Supplement: Extended Data Figure 1-2 — Confirmation of optic fiber location and expression specificity of Group I. A, Optic fiber placement for D2-eYFP (grey) and D2-ChR2 (blue) rats (nD2-eYFP = 7; nD2-ChR2 = 10). B, Number of D2R+ and eYFP+ cells per area as evaluated by IF. Almost all of eYFP+ cells are also D2R+, confirming the specificity of the construct. C, Number of D1R+ and eYFP+ cells per area. D, Number of ChAT+ and eYFP+ cells. Only a few D1R+ and ChAT+ cells express the construct (nD2-eYFP = 6; nD2-ChR2 = 6). Error bars denote SEM. Download Figure 1-2, TIF file. [file sup_enu-eN-NWR-0386-18-s03.tif]

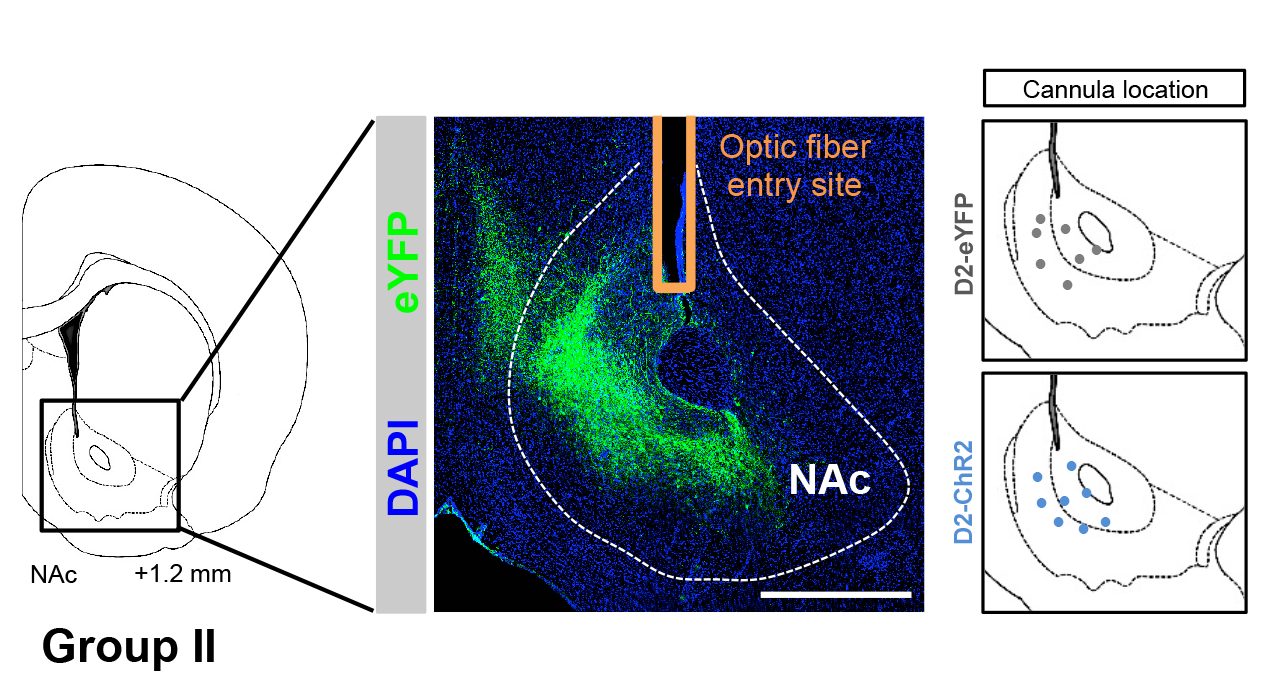

Supplement: Extended Data Figure 2-1 — Representative image of viral infection extent and cannula entry site (numbers represent distance to bregma; scale bar: 1 mm); optic fiber placement for D2-eYFP (grey) and D2-ChR2 (blue) rats of Group II (nD2-eYFP = 7; nD2-ChR2 = 8). Download Figure 2-1, TIF file. [file sup_enu-eN-NWR-0386-18-s04.tif]

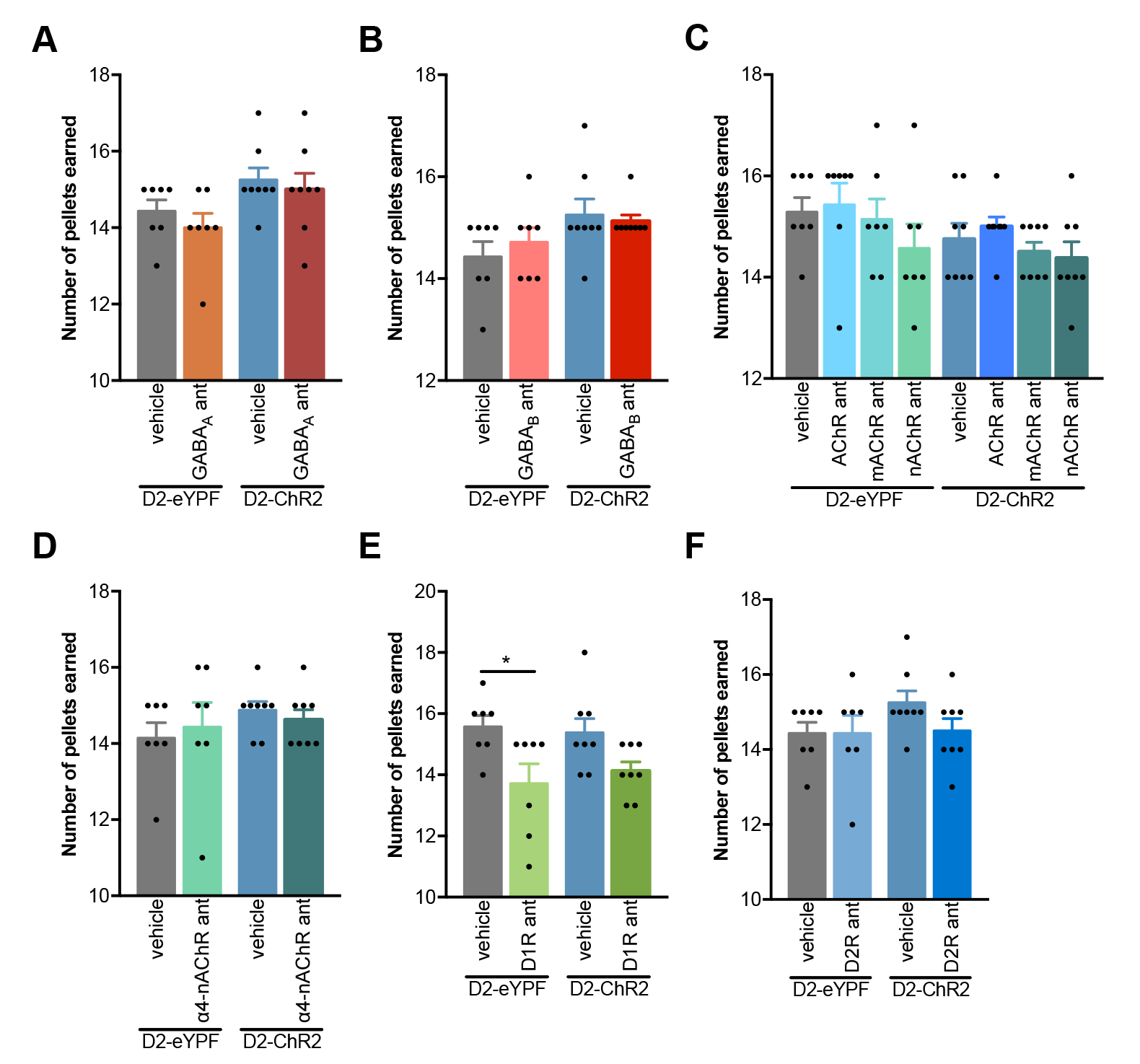

Supplement: Extended Data Figure 2-2 — Number of pellets consumed during the PR session with optical stimulation with previous administration of different antagonists. A, GABAA receptor antagonist (bicuculline, 75 ng). B, GABAB receptor antagonist (GCP-55845, 44 ng). C, mAChR antagonist (scopolamine, 25 μg) + nAChR antagonist (mecamylamine, 22.5 μg). D, α4-nAChR antagonist (DHβE, 0.7 μg). E, D1R antagonist (SCH-23390, 0.25 μg). F, D2R antagonist (sulpiride, 0.2 μg). Error bars denote SEM; *p < 0.05. Download Figure 2-2, TIF file. [file sup_enu-eN-NWR-0386-18-s05.tif]

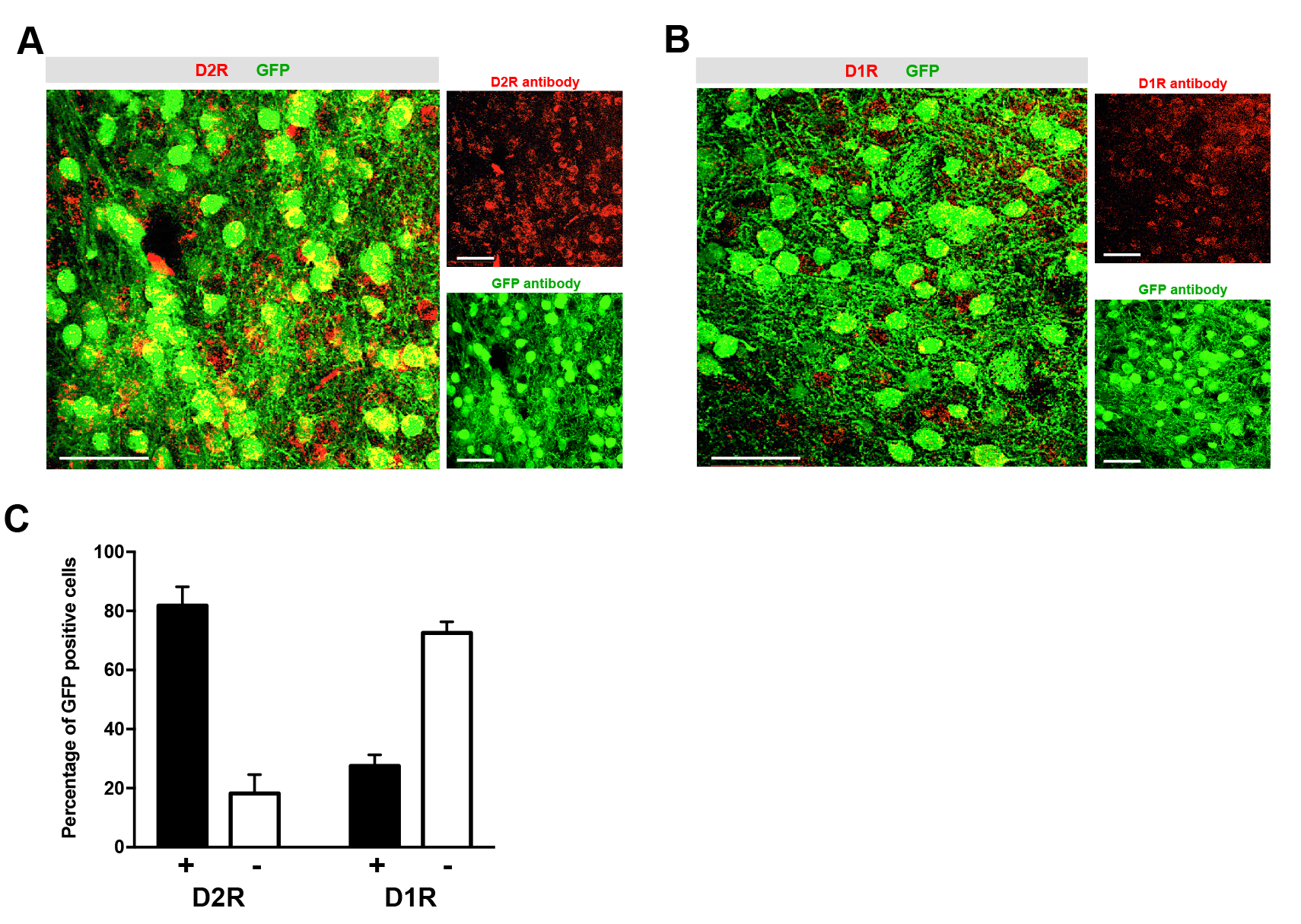

Supplement: Extended Data Figure 3-1 — IF against GFP and D1R or D2R in D2-EGFP reporter strain. A, Representative image of a section of a D2-GFP animal labelled with anti-GFP and anti-dopamine receptor D2 (scale bar: 50 μm). B, Representative image of a section of a D2-GFP animal labelled with anti-GFP and anti-D1R (scale bar: 50 μm). C, Respective quantification of IF; 54.4% of total cells were GFP+, in agreement with half of the NAc cells being D2-MSNs. Of those GFP+ cells, 83% were D2R+ and 17% D2R-; whereas most (73%) of these cells were D1R-. Error bars denote SEM. Download Figure 3-1, TIF file. [file sup_enu-eN-NWR-0386-18-s06.tif]

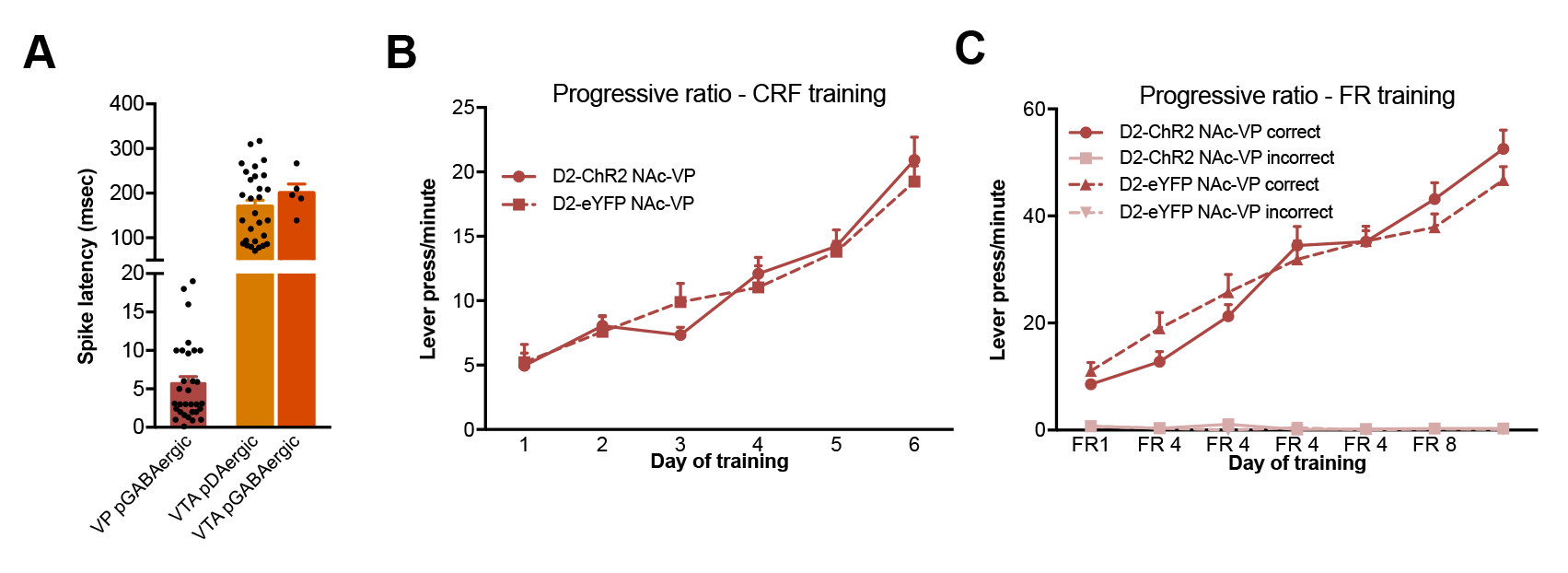

Supplement: Extended Data Figure 4-1 — Additional data from optogenetic activation experiments. A, Spike latency in the VP and VTA neurons in response to NAc D2-MSN optogenetic stimulation. VP neurons present reduced spike latency to fire, consistent with a monosynaptic input from D2-MSNs, whereas VTA neurons present spike latencies indicative of polysynaptic modulation. B,C, CRF and FR learning curves of D2-eYFP and D2-ChR2 NAc-VP animals. Download Figure 4-1, TIF file. [file sup_enu-eN-NWR-0386-18-s07.tif]
